# Association Between Federal Value-Based Incentive Programs and Health Care–Associated Infection Rates in Safety-Net and Non–Safety-Net Hospitals

**DOI:** 10.1001/jamanetworkopen.2020.9700

**Published:** 2020-07-08

**Authors:** Heather E. Hsu, Rui Wang, Carly Broadwell, Kelly Horan, Robert Jin, Chanu Rhee, Grace M. Lee

**Affiliations:** 1 Department of Pediatrics, Boston University School of Medicine, Boston, Massachusetts; 2 Department of Population Medicine, Harvard Pilgrim Health Care Institute, Harvard Medical School, Boston, Massachusetts; 3 Department of Biostatistics, Harvard T.H. Chan School of Public Health, Boston, Massachusetts; 4 Department of Medicine, Brigham and Women’s Hospital, Boston, Massachusetts; 5 Department of Pediatrics, Stanford University School of Medicine, Palo Alto, California

## Abstract

**Importance:**

In the US, federal value-based incentive programs are more likely to penalize safety-net institutions than non–safety-net institutions. Whether these programs differentially change the rates of targeted health care–associated infections in safety-net vs non–safety-net hospitals is unknown.

**Objective:**

To assess the association of Hospital-Acquired Condition Reduction Program (HACRP) and Hospital Value-Based Purchasing (HVBP) implementation with changes in rates of targeted health care–associated infections and disparities in rates among safety-net and non–safety-net hospitals.

**Design, Setting, and Participants:**

This interrupted time series included all US acute care hospitals enrolled in the Preventing Avoidable Infectious Complications by Adjusting Payment study that participated in mandatory reporting to the National Healthcare Safety Network from January 1, 2013, through June 30, 2018. Hospital characteristics were obtained from the 2015 American Hospital Association annual survey. Penalty statuses for 2015 to 2018 were obtained from Hospital Compare. Data were analyzed between July 9, 2018, and October 1, 2019.

**Exposures:**

HACRP and HVBP implementation in fiscal year 2015 or 2016.

**Main Outcomes and Measures:**

The primary outcomes were rates of 4 health care–associated infections: central line–associated bloodstream infection (CLABSI), catheter-associated urinary tract infection (CAUTI), surgical site infection (SSI) after colon surgical procedures, and SSI after abdominal hysterectomy procedures. Regression models were fit using generalized estimating equations to assess the association of HACRP and HVBP implementation with health care–associated infection rates and disparities in infection rates.

**Results:**

Of the 618 acute care hospitals included in this study, 473 (76.5%) were non–safety net and 145 (23.5%) were considered safety net. In these hospitals, HACRP and HVBP implementation was not associated with improvements in level or trend for any health care–associated infection examined (eg, CAUTI in safety-net hospitals: incidence rate ratio [IRR] for level change, 0.98 [95% CI, 0.79-1.23; *P* = .89]; IRR for change in slope, 1.00 [95% CI, 0.97-1.03; *P* = .80]). Before program implementation, infection rates were statistically significantly higher for safety-net than for non–safety-net hospitals for CLABSI (IRR, 1.23; 95% CI, 1.07-1.42; *P* = .004), CAUTI (IRR, 1.38; 95% CI, 1.16-1.64; *P* < .001), and SSI after colon surgical procedure (odds ratio [OR], 1.26; 95% CI, 1.06-1.50; *P* = .009). The disparity persisted over time when comparing the last year of the study with the first year (CLABSI: ratio of ratios [ROR], 0.93 [95% CI, 0.77-1.13; *P* = .48]; CAUTI: ROR, 0.90 [95% CI, 0.73-1.10; *P* = .31]; SSI after colon surgical procedures: ROR, 0.96 [95% CI, 0.78-1.20; *P* = .75]). Rates of SSI after abdominal hysterectomy procedure were similar in safety-net and non–safety-net hospitals before implementation (OR, 1.13; 95% CI, 0.91-1.40; *P* = .27) but higher after implementation (OR, 1.43; 95% CI, 1.11-1.83; *P* = .006), although this change was not significant (ROR, 1.20; 95% CI, 0.91-1.59; *P* = .20).

**Conclusions and Relevance:**

This study found that HACRP and HVBP implementation was not associated with any improvements in targeted health care–associated infections among safety-net or non–safety-net hospitals or with changes in disparities in infection rates. Given the persistent health care–associated infection rate disparities, these programs appear to function as a disproportionate penalty system for safety-net hospitals that offer no measurable benefits for patients.

## Key Points

**Question:**

How is the implementation of federal value-based incentive programs associated with disparities in health care–associated infection rates in safety-net and non–safety-net hospitals in the US?

**Findings:**

In this interrupted time series study of 618 hospitals, implementation of federal value-based incentive programs was not associated with any improvements in targeted health care–associated infection rates or with changes in disparities in infection rates among safety-net and non–safety-net hospitals.

**Meaning:**

Results of this study suggest that, given the persistent disparities in health care–associated infection rates, value-based incentive programs currently function as a disproportionate financial penalty system for safety-net hospitals that provide no measurable population-level benefits.

## Teaser

This interrupted time series study evaluates the association between federal value-based incentive programs and health care–associated infection rates in acute care hospitals in the United States.

## Introduction

Health care–associated infections represent an important target of efforts in the United States to connect health care quality to payment. The Affordable Care Act established 2 value-based incentive programs that link hospitals’ health care–associated infection rates with financial incentives and penalties: the Hospital-Acquired Condition Reduction Program (HACRP) and the Hospital Value-Based Purchasing (HVBP) program, which includes health care–associated infection metrics in its safety domain.^1^ In October 2014, these programs began comparing hospital performance on selected infection metrics with national benchmarks based on prospective infection surveillance data reported to the National Healthcare Safety Network (NHSN) of the Centers for Disease Control and Prevention. At present, HVBP rewards or penalizes the highest- and lowest-performing hospitals by up to 2% of the total inpatient payments the hospital received, whereas the HACRP reduces payments by up to 1% for the lowest performers.^1,2^

Previous studies have shown worse outcomes in many targeted quality measures adopted by federal value-based incentive programs among patients with low income and among those who identify as members of racial/ethnic minority groups compared with patients without socioeconomic disadvantages.^3,4,5,6,7,8^ Safety-net hospitals that care for a higher proportion of patients with low socioeconomic status also tend to demonstrate worse performance, even after accounting for case mix.^9^ As a result, safety-net institutions are more likely than non–safety-net facilities to incur financial penalties,^9,10,11,12,13^ which may have potential implications for the patients served.^3,14,15,16^ In the case of health care–associated infections, the disproportionate assessment of financial penalties for safety-net institutions could be a factor in worsening financial hardships and subsequently disparities affecting these hospitals. However, it remains unclear whether financial penalty policies are associated with population-level improvements in infection rates or with differential implications for infection rates among safety-net vs non–safety-net hospitals. Therefore, the objective of this study was to examine whether HACRP and HVBP implementation was associated with changes in trends in targeted health care–associated infection rates or with disparities in health care–associated infection rates between safety-net and non–safety-net hospitals.

## Methods

### Study Design, Population, and Data Sources

This study had an interrupted time series design. Prospective NHSN surveillance data were obtained for a cohort of nonfederal acute care hospitals subject to the Inpatient Prospective Payment System (IPPS) and enrolled in the Preventing Avoidable Infectious Complications by Adjusting Payment (PAICAP) study.^17^ The NHSN is the most comprehensive surveillance system in the US and is used by more than 4000 acute care facilities in all 50 states, Washington, DC, and Puerto Rico.^18^ Hospitals prospectively track reportable conditions using standardized NHSN surveillance case definitions for health care–associated infections based on laboratory and clinical data.^19^ All hospitals in the PAICAP study that participated in mandatory NHSN reporting from January 1, 2013, through June 30, 2018, were included. The institutional review board of Harvard Pilgrim Health Care Institute approved the present study and provided a waiver of informed consent because the study used deidentified data. We followed the Strengthening the Reporting of Observational Studies in Epidemiology (STROBE) reporting guideline.

Hospital characteristics were obtained from the 2015 American Hospital Association annual survey,^20^ including hospital size (<100, 100-399, or ≥400 beds), teaching status (graduate, major, minor, or nonteaching), ownership type (public, for profit, or not for profit), location (metropolitan, micropolitan, or rural), and proportion of Medicare- or Medicaid-covered inpatient days. The HACRP penalty statuses for 2015 to 2018 were obtained from Hospital Compare.^21^ In the primary analyses, safety-net hospitals were defined using the Disproportionate Share Hospital patient percentage (DSH index) from the 2018 Centers for Medicare and Medicaid Services Historical Impact File.^22^ Safety-net hospitals were identified as those with DSH indices in the highest quartile of all hospitals under IPPS that responded to the 2015 American Hospital Association survey,^23^ whereas those with DSH indices in the first through third quartiles were considered to be non–safety-net hospitals. For hospitals without a DSH index (n = 25 [4%]), safety-net hospitals were defined as those in the top quartile for proportion of Medicaid-covered inpatient days among all hospitals under IPPS that responded to the American Hospital Association survey.

### Outcomes

We examined changes in trends of 4 health care–associated infection outcomes targeted by HACRP and HVBP: device-associated central line–associated bloodstream infection (CLABSI; infections per 1000 central line–days), catheter-associated urinary tract infection (CAUTI; infections per 1000 indwelling urinary catheter–days), complex surgical site infection (SSI) after colon surgical procedure (infections per 100 procedures), and complex SSI after abdominal hysterectomy (infections per 100 procedures). For CLABSI and CAUTI, only cases reported from adult critical care units participating in mandated reporting of these outcomes since January 2011 for CLABSI and 2012 for CAUTI were included because reporting from additional units did not become mandatory until January 2015. Per NHSN protocol, complex SSIs were defined as deep incisional primary or organ space SSIs that occurred within 30 days of the procedure.

Three health care–associated infection outcomes (CLABSI, complex SSI after colon surgical procedure, and complex SSI after abdominal hysterectomy) were defined with the NHSN standardized surveillance case definitions that were in use at the time that each case was reported. For CAUTI, the analyses were restricted to only those cases associated with urine cultures growing 100 000 or more colony-forming units of bacterial organisms per milliliter to account for a January 2015 NHSN case definition change that excluded cases associated with urine cultures growing less than 100 000 colony-forming units of bacteria or nonbacterial organisms.^24,25^ Additional January 2015 revisions in NHSN case definitions and procedures affecting the other 3 outcomes^24^ were accounted for in the study’s interrupted time series design. These revisions included more stringent criteria for the designation of bloodstream infections secondary to sources other than central lines, which were associated with observed increases in CLABSI rates,^26,27,28^ and exclusion of pediatric cases and infections present at the time of surgical procedure, which were associated with observed decreases in SSI rates.^29^

### Accounting for Differential Timing of HACRP and HVBP Implementation and NHSN Definition Revisions

All NHSN revisions to case definitions affecting health care–associated infection outcomes occurred in January 2015. However, the timing of HACRP and HVBP implementation differed for each outcome examined. Definition revisions and implementation timing were accounted for in the interrupted time series design as follows.

For CLABSI, both HACRP and HVBP implementation began in fiscal year 2015 (October 1, 2014). Given the proximity of the HACRP and HVBP implementation and the NHSN revisions, the level and slope of quarterly CLABSI rates were assessed before and after a 2-quarter transition period, which was defined a priori to be inclusive of the implementation beginning October 1, 2014, and the revisions on January 1, 2015. The CLABSI events during this transition period were not included in the analyses.

For CAUTI, HACRP implementation began in fiscal year 2015 and HVBP implementation began in fiscal year 2016 (October 1, 2015). As such, the level and slope of quarterly CAUTI rates were assessed before and after a program implementation transition period.^30^ This transition period was defined a priori to include the 1-year lag between implementation of HACRP and HVBP for CAUTI (October 1, 2014, to September 30, 2015), and CAUTI outcomes during this period were not included in the analyses. Because NHSN revisions for CAUTI were accounted for by restricting the cases included in the analyses to only those that met the updated 2015 case definition, the revisions did not require an additional node in the time series.

For the SSIs, both HACRP and HVBP implementation began in fiscal year 2016. Therefore, the level and the slope of quarterly rates of SSIs after colon surgical procedure or abdominal hysterectomy were assessed before and after the NHSN revision (January 1, 2015) as well as the HACRP and HVBP implementation (October 1, 2015).

### Statistical Analysis

Data were analyzed between July 9, 2018, and October 1, 2019. We used χ^2^, Fisher exact, or Wilcoxon rank sum tests to explore the differences in hospital characteristics by safety-net status. In the primary analyses, we fit regression models to assess for changes in either level or trend of health care–associated infections after HACRP and HVBP implementation and NHSN case definition revisions when applicable. We fit negative binomial models for quarterly CLABSI and CAUTI counts with device-days in the offset term and logistic regression models for the proportion of colon or abdominal hysterectomy surgical procedures, resulting in an SSI per quarter. Health care–associated infection counts and proportions were aggregated to the quarterly level to increase the stability of the estimates. We used generalized estimating equations with robust sandwich variance estimators to account for possible clustering at the hospital and unit levels.

All models included time (to model secular trends), an indicator of hospital safety-net status, an indicator of the postimplementation period (allowing for evaluation of an immediate change after program implementation), and 2- and 3-way interaction terms of all of these variables to ascertain whether program implementation was associated with a change in level or trends of outcomes among safety-net vs non–safety-net hospitals. Models for SSI outcomes included an additional indicator of the post-NHSN case definition revision period and relevant interaction terms. To quantify whether disparities in health care–associated infection rates between safety-net and non–safety-net hospitals changed after program implementation, we calculated mean incidence rate ratios (IRRs) or odds ratios (ORs) for each outcome for both safety-net and non–safety-net hospitals during the first year (January 1, 2013, to December 31, 2013) and last year (July 1, 2017, to June 30, 2018) of the present study. Ratios of these ratios (RORs) were then calculated to compare the disparity in health care–associated infection rates between safety-net and non–safety-net hospitals in the last year with the first year of study (post- vs pre-HACRP/HVBP implementation). An ROR that was significantly less than 1 indicated that the disparity narrowed in the postimplementation period, whereas an ROR that was significantly greater than 1 indicated that the disparity widened. Two-sided *P* < .05 was considered to be statistically significant. Analyses were performed in SAS, version 9.4 (SAS Institute).

We conducted sensitivity analyses that (1) included only hospitals that contributed data in both the first and last study years (consistent reporters); (2) included only hospitals with an available DSH index to define the safety-net status; (3) used a more sensitive definition of safety-net status, that is, those hospitals with a percentage of Medicaid-covered inpatient days in the top quartile of all hospitals under IPPS; and (4) included additional hospital characteristics as covariates in the model that were significantly different between safety-net and non–safety-net hospitals but were not intimately associated with the definition of safety-net status, such as region, hospital size (≥400 or <400 beds), and teaching status.

## Results

### Study Population

Between January 1, 2013, and June 30, 2018, a total of 618 hospitals in the PAICAP study located in 49 states and Washington, DC, met IPPS criteria and reported to NHSN cases of CLABSI, CAUTI, and/or SSI after colon surgical procedures or abdominal hysterectomy procedures. Of the 618 hospitals, 473 (76.5%) were non–safety net and 145 (23.5%) were considered safety net. Most hospitals contributed to health care–associated infection outcome data in both the first and last years of the present study (CLABSI: 560 of 602 hospitals [93.0%]; CAUTI: 552 of 592 hospitals [93.2%]; SSI after colon surgical procedure: 567 of 606 hospitals [93.6%]; SSI after abdominal hysterectomy: 538 of 598 hospitals [90.0%]). Significant differences between safety-net and non–safety-net hospitals included greater use of central lines (median interquartile range [IQR], 0.48 [0.37-0.58] vs 0.45 [0.33-0.54]; *P* = .02) and a higher volume of abdominal hysterectomy procedures (median [IQR], 602 [202-1159] vs 363 [106-882]; *P* = .002) in safety-net hospitals as well as a higher proportion of safety-net hospitals with more than 400 beds (62 of 145 [42.8%] vs 86 of 473 [18.2%]; *P* < .001), public ownership (22 of 145 [15.2%] vs 23 of 473 [4.9%]; *P* < .001), and designation as a major teaching hospital (44 of 145 [30.3%] vs 49 of 473 [10.4%]; *P* < .001) (Table 1). In addition, a greater proportion of safety-net hospitals compared with non–safety-net hospitals (84 of 134 [62.7%] vs 225 of 452 [49.8%]; *P* = .009) received a HACRP financial penalty in 1 year or more.

**Table 1.** Characteristics of National Healthcare Safety Network Study Hospitals by Safety-Net Status

Characteristic	Safety-net hospitals (n = 145)	Non–safety-net hospitals (n = 473)	*P* value^a^
Outcome reported, No. (%)			
CLABSI	143 (98.6)	459 (97.0)	.38
CAUTI	140 (96.6)	452 (95.6)	.81
SSI after colon surgical procedure	139 (95.9)	467 (98.7)	.04
SSI after abdominal hysterectomy	138 (95.2)	460 (97.3)	.28
DUR, median (IQR)			
Central line	0.48 (0.37-0.58)	0.45 (0.33-0.54)	.02
Indwelling urinary catheter	0.60 (0.52-0.67)	0.60 (0.51-0.67)	.74
Quarterly procedural volume, median (IQR)			
Colon surgical procedure	530 (290-1011)	475 (200-930)	.20
Abdominal hysterectomy	602 (202-1159)	363 (106-882)	.002
Region, No. (%)			
Midwest	24 (16.6)	91 (19.2)	.03
Northeast	38 (26.2)	140 (29.6)	
South	47 (32.4)	175 (37.0)	
West	36 (24.8)	67 (14.2)	
Location, No. (%)			
Metropolitan	130 (89.7)	401 (84.8)	.11
Micropolitan	14 (9.7)	53 (11.2)	
Rural	1 (0.7)	19 (4.0)	
Hospital size, No. (%), beds			
<100	11 (7.6)	89 (18.8)	<.001
100-399	72 (49.7)	298 (63.0)	
≥400	62 (42.8)	86 (18.2)	
Ownership type, No. (%)			
For profit	46 (31.7)	150 (31.7)	<.001
Not for profit	77 (53.1)	300 (63.4)	
Public	22 (15.2)	23 (4.9)	
Teaching status, No. (%)^b^			
Graduate teaching	53 (36.6)	180 (38.1)	<.001
Major teaching	44 (30.3)	49 (10.4)	
Minor teaching	3 (2.1)	23 (4.9)	
Nonteaching	45 (31.0)	221 (46.7)	
FTE nurses, median No. per 100 patient-days (IQR)	0.75 (0.60-0.95)	0.81 (0.65-1.00)	.06
% Inpatient-days covered by Medicare, median (IQR)	43.0 (37.2-48.4)	52.6 (46.9-61.2)	<.001
% Inpatient-days covered by Medicaid, median (IQR)	28.7 (24.5-35.5)	18.1 (12.2-22.5)	<.001
Received HACRP financial penalty in ≥1 y, No. (%)^c^	84 (62.7)	225 (49.8)	.009

Abbreviations: CAUTI, catheter-associated urinary tract infection; CLABSI, central line–associated bloodstream infection; DUR, device utilization ratio (device-days per patient-days); FTE, full-time equivalent; HACRP, Hospital-Acquired Condition Reduction Program; IQR, interquartile range; SSI, surgical site infection.

^a^ *P* values for categorical characteristics were calculated using χ^2^ or Fisher exact test as appropriate. *P* values for continuous characteristics were calculated using Wilcoxon rank sum test.

^b^ All hospitals were placed into 1 of 4 categories based on their response to the American Hospital Association survey: major teaching hospitals (members of the Council of Teaching Hospitals and Health Systems), graduate teaching hospitals (nonmembers with a residency training program approved by the Accreditation Council for Graduate Medical Education), minor teaching hospitals (nonmembers with a medical school affiliation reported to the American Medical Association), and nonteaching hospitals (all other institutions).

^c^ HACRP financial penalty status was available for 134 (92%) safety-net hospitals and 452 (96%) non–safety-net hospitals for the years 2015 to 2018.

### Association of Program Implementation With Changes in Infection Rates Within and Between Safety-Net and Non–Safety-Net Hospitals

The Figure shows observed and estimated rates of each outcome examined over time for safety-net and non–safety-net hospitals. No significant differences were observed between safety-net and non–safety-net hospitals in immediate level or slope changes in health care–associated infection rates at the time of HACRP or HVBP implementation (eg, postimplementation SSI after colon surgical procedure: OR for level change, 2.02 [95% CI, 0.55-7.40; *P* = .29]; OR for slope change, 0.93 [95% CI, 0.81-1.07; *P* = .30]) and/or NHSN definition revisions (eg, postdefinition revision SSI after colon surgical procedures: OR for level change, 0.43 [95% CI, 0.12-1.53; *P* = .19]; OR for slope change, 1.08 [95% CI, 0.94-1.24; *P* = .26]) for any outcomes examined (eTable 1 in the Supplement).

**Figure.**
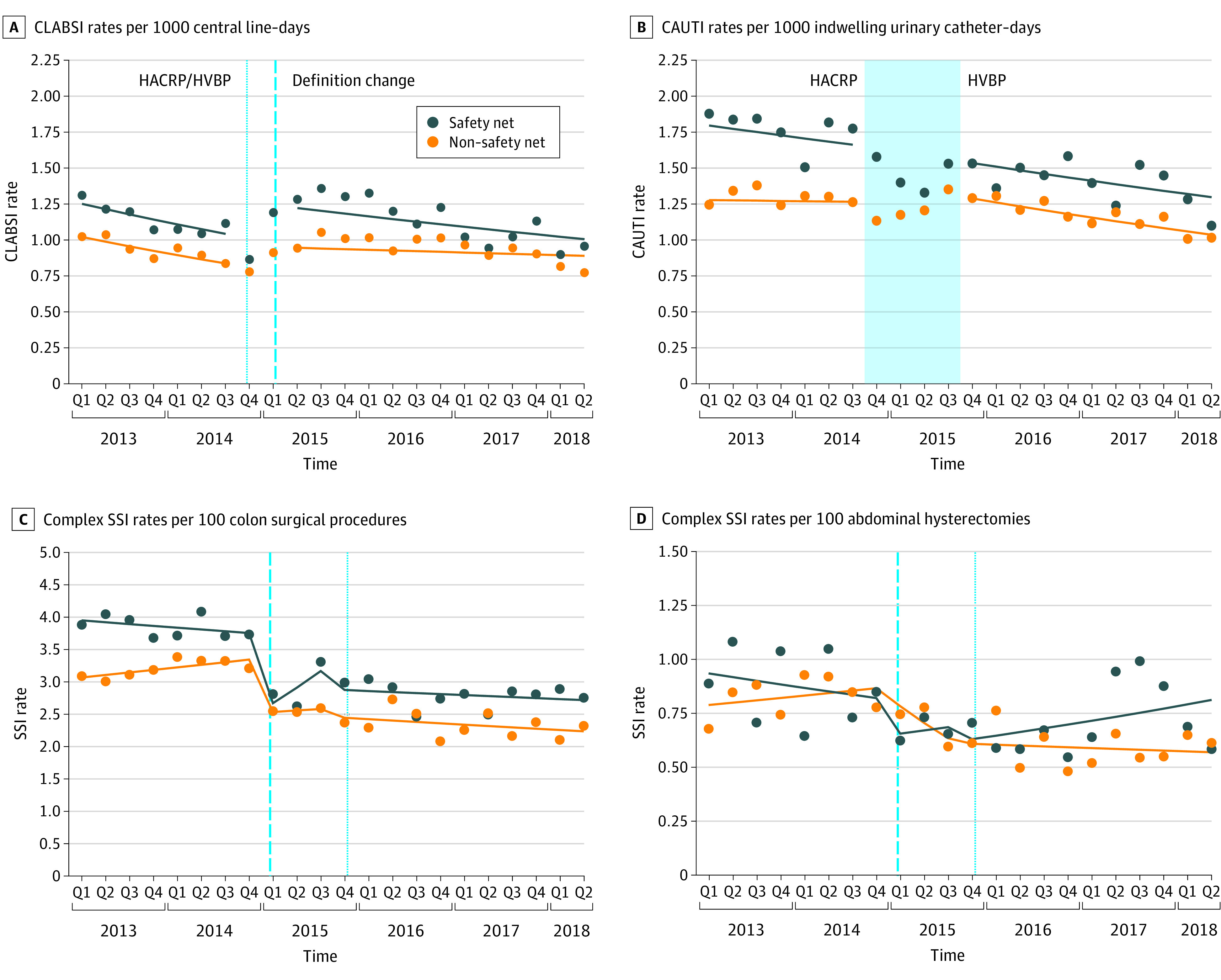
Observed and Estimated Health Care–Associated Infection Rates Over Time by Hospital Safety-Net Status Each panel depicts observed and estimated rates of all Hospital-Acquired Condition Reduction Program (HACRP)/Hospital Value-Based Purchasing (HVBP)-targeted health care–associated infections aggregated for safety-net and non–safety-net hospitals by quarter (Q). Circles indicate observed rates, and lines indicate model-estimated rates. In panels A, C, and D, the vertical short-dash line indicates timing of HACRP and HVBP implementation, and the vertical long-dash line indicates timing of the National Healthcare Safety Network surveillance case definition revisions in January 2015. In panel B, the wider shaded area represents the period between onset of HACRP penalties and onset of HVBP penalties or incentive payments for catheter-associated urinary tract infection (CAUTI) rates, as these programs were not implemented simultaneously for this outcome. CLABSI indicates central line–associated bloodstream infection; SSI, surgical site infection.

Within safety-net and non–safety-net hospitals, HACRP and HVBP implementation was not associated with changes in level or trend for CAUTIs (eg, in safety-net hospitals: IRR for level change, 0.98 [95% CI, 0.79-1.23; *P* = .89]; IRR for change in slope, 1.00 [95% CI, 0.97-1.03; *P* = .80]) or the SSIs (eg, after colon procedures in safety-net hospitals: OR for level change, 2.23; 95% CI, 0.81-6.65; *P* = .12). Significant immediate increases in CLABSI rates of 25% for safety-net hospitals (IRR, 1.25; 95% CI, 1.04-1.49; *P* = .02) and 18% for non–safety-net hospitals (IRR, 1.18 [95% CI, 1.04-1.34; *P* = .01) were observed at the time of the coincident NHSN definition revision and HACRP and HVBP implementation as well as a significant increase in the slope of the trend among non–safety-net hospitals (IRR, 1.03; 95% CI, 1.00-1.05; *P* = .03) (eTable 1 in the Supplement).

### Disparities in Health Care–Associated Infection Rates

Rates of CLABSI (IRR, 1.23; 95% CI, 1.07-1.42; *P* = .004), CAUTI (IRR, 1.38; 95% CI, 1.16-1.64; *P* < .001), and SSI after colon surgical procedures (OR, 1.26; 95% CI, 1.06-1.50; *P* = .009) were significantly higher for safety-net than for non–safety-net hospitals in the first year of study, before HACRP and HVBP implementation and the 2015 NHSN case definition revisions (Table 2). For these outcomes, the disparity in health care–associated infection rates between safety-net and non–safety-net hospitals persisted when comparing the last vs the first year of study (CLABSI: ROR, 0.93 [95% CI, 0.77-1.13; *P* = .48]; CAUTI: ROR, 0.90 [95% CI, 0.73-1.10; *P* = .31]; SSI after colon surgical procedures: ROR, 0.96 [95% CI, 0.78-1.20; *P* = .75]). Rates of SSI after abdominal hysterectomy procedures were similar in the safety-net and non–safety-net hospitals during the first year (OR, 1.13, 95% CI, 0.91-1.40; *P* = .27) but were statistically significantly higher during the last year (OR, 1.43; 95% CI, 1.11-1.83; *P* = .006). However, because of the small number of events, there was insufficient power to detect the emergence of a statistically significant disparity when comparing the preimplementation with the postimplementation period (ROR, 1.20; 95% CI, 0.91-1.59; *P* = .20).

**Table 2.** Comparison of the Disparity in Health Care–Associated Infection Rates Between Safety-Net and Non–Safety-Net Hospitals Before and After VBIP Implementation

Outcome	Pre-VBIP^a^ disparity		Post-VBIP^a^ disparity		Post- vs pre-VBIP comparison	
	Mean IRR or OR (95% CI)^b^	*P* value	Mean IRR or OR (95% CI)	*P* value	ROR (95% CI)	*P* value
CLABSI per 1000 central line–days	1.23 (1.07-1.42)	.004	1.15 (1.00-1.32)	.046	0.93 (0.77-1.13)	.48
CAUTI per 1000 catheter-days	1.38 (1.16-1.64)	<.001	1.24 (1.05-1.47)	.01	0.90 (0.73-1.10)	.31
SSI per 100 colon surgical procedures	1.26 (1.06-1.50)	.009	1.22 (1.03-1.43)	.02	0.96 (0.78-1.20)	.75
SSI per 100 abdominal hysterectomy procedures	1.13 (0.91-1.40)	.27	1.43 (1.11-1.83)	.006	1.20 (0.91-1.59)	.20

Abbreviations: CAUTI, catheter-associated urinary tract infection; CLABSI, central line–associated bloodstream infection; IRR, incident rate ratio; OR, odds ratio; ROR, ratio of ratios; SSI, surgical site infection; VBIP, value-based incentive program.

^a^ The pre-VBIP implementation period included data from January 1, 2013, through December 31, 2013. The post-VBIP period included data from July 1, 2017, through June 30, 2018.

^b^ IRRs are reported for CLABSI and CAUTI rates. ORs are reported for the SSIs.

### Sensitivity Analyses

The results of sensitivity analyses were consistent with those of the primary analyses, indicating a lack of association between HACRP and HVBP implementation and changes in disparities in health care–associated infection rates between safety-net and non–safety-net hospitals (eTable 2 in the Supplement). For CLABSI, the results of the sensitivity analyses were also consistent with those of the main analyses (eg, postimplementation vs preimplementation rates among consistent reporters: ROR, 0.93; 95% CI, 0.77-1.13; *P* = .49).

## Discussion

In this interrupted time series study of 618 acute care hospitals, HACRP and HVBP implementation was generally not associated with increases or decreases in rates of targeted health care–associated infections among safety-net and non–safety-net hospitals. Increases in CLABSI rates observed at the time of program implementation were similar for both safety-net and non–safety-net hospitals and were most likely associated with coincident NHSN case definition revisions.^26,27,28^ The present study also demonstrated the persistent disparities in reported health care–associated infection rates between safety-net and non–safety-net hospitals, which neither significantly improved nor worsened after HACRP and HVBP implementation.

The reason for health care–associated infection rate disparities is not completely understood and not eliminated by current methods of adjustment for clinical risk factors or case mix.^9^ The reason for the disparities is likely multifactorial and associated with a complex mix of health care environmental factors and patient-related clinical and social factors,^31,32,33^ including the adverse effects of structural racism, discrimination, and toxic stress among patients disproportionately served by safety-net institutions such as those with low income and/or those who identify as members of racial/ethnic minority groups.^34,35,36,37^

Whatever their cause, these disparities in health care–associated infection rates contribute to the disproportionate representation of safety-net hospitals among penalized institutions^11,13^ and may have unintended consequences for the financial stability of the safety net and the quality of health care for the patients served. Although Medicaid expansion under the Affordable Care Act reduced uncompensated care costs for many safety-net hospitals,^38,39^ these hospitals continue to have low operating margins and often rely on nonclinical sources of revenue^40^ or state and federal funds, particularly DSH payments,^41^ to offset financial losses associated with remaining uncompensated care and Medicaid reimbursement that is below actual costs.^42,43^ For example, in 2017, the mean operating margin for members of the safety-net hospital organization America’s Essential Hospitals was 1.6% compared with 7.8% for all hospitals nationwide. The disproportionate assessment of financial penalties for safety-net hospitals is particularly relevant given the reductions in DSH payments for safety-net hospitals planned for fiscal year 2020, which were projected to be $4 billion for 2020 and $8 billion for each subsequent year through 2025.^44,45^ Without these DSH payments, the mean operating margin for safety-net institutions is anticipated to decrease to –3.0%.^41^

To improve equity in the HACRP and HVBP, the Centers for Medicare and Medicaid Services could consider redesigning the penalty structure for these programs. Currently, neither program incorporates any form of social risk factor adjustment prior to assessing incentive payments or penalties. Given that the same group of health care–associated infection metrics is included in both programs, the disparities between safety-net and non–safety-net hospitals for health care–associated infections may result in penalization from both programs for more safety-net hospitals than non–safety-net hospitals. Potential policy-level improvements may include the elimination of double counting of health care–associated infection metrics in the 2 programs and the stratification of hospitals according to safety-net status before penalty assessment.

Various potential approaches to social risk factor adjustment exist, many of which present challenges with data availability or risk of establishing a lower quality threshold for safety-net hospitals.^46,47^ However, social risk factor adjustment is not without precedent among value-based incentive programs. For example, after disparities were noted in hospital readmissions,^10,12,48^ the Hospital Readmissions Reduction Program adopted a form of social risk factor adjustment that was applied for the first time in fiscal year 2019.^49^ The new system evaluates each hospital’s performance on readmissions compared with performance by other hospitals with similar proportions of patients eligible for both Medicare and Medicaid. By stratifying hospitals on the basis of this proxy measure for social risk, the Hospital Readmissions Reduction Program hopes to avoid systematic penalization of safety-net hospitals and the pitfall of setting lower quality benchmarks for these institutions.

### Strengths and Limitations

Strengths of this study include the use of longitudinal health care–associated infection surveillance data from approximately 20% of all US acute care hospitals participating in HACRP and HVBP. More than 90% of study hospitals contributed data consistently, and all hospitals participated in mandated NHSN health care–associated infection reporting for at least 1 year before the study’s start date, mitigating the risk of ascertainment and selection bias. In addition, our finding of the lack of association between HACRP and HVBP implementation and changes in health care–associated infection disparities among safety-net and non–safety-net hospitals was consistent across the multiple outcomes examined and sensitivity analyses conducted, and it accounted for co-occurring NHSN definition revisions when possible.

This study has several limitations. First, it examined a selection of HACRP- and HVBP-targeted health care–associated infection outcomes. Nontargeted outcomes were not included because of the risk of ascertainment bias associated with the lack of mandated reporting. Additional targeted outcomes, including hospital-associated *Clostridioides* (formerly *Clostridium*) *difficile* infection and methicillin-resistant *Staphylococcus aureus* bloodstream infection, were not included because they were not adopted as targets until fiscal year 2017.

Second, the co-occurring NHSN definition revisions and timing of HACRP and HVBP implementation for CLABSI made it impossible to disentangle the particular contribution of program implementation alone to changes in CLABSI rates. However, smaller medical record review–based studies suggest that increases in CLABSI observed at the time of HACRP and HVBP implementation were most likely associated with coincident NHSN definition revisions.^26,27,28^

Third, this study used health care–associated infection rates as the primary outcomes rather than standardized infection ratios, which are the risk-adjusted observed-to-estimated summary measures based on the health care–associated infection rates that are used to derive HACRP and HVBP performance scores. Use of rates was necessary to account for NHSN case definition revisions during the study period.

## Conclusions

In this interrupted time series study, HACRP and HVBP implementation did not appear to be associated with any improvements in targeted health care–associated infection rates among safety-net or non–safety-net hospitals or with changes in disparities in infection rates between these hospitals. Given the persistent disparities, these value-based incentive programs currently function as a disproportionate penalty system for safety-net hospitals that provide no measurable population-level benefits. To explicitly address equity, the Centers for Medicare and Medicaid Services should consider redesigning these programs’ penalty structures to ensure that the safety-net health care systems have adequate support to stimulate meaningful improvements.

## Notes

Supplement. **eTable 1.** Association of Value-Based Incentive Program Implementation and Health Care–Associated Infection Case Definition Changes on Reported Quarterly Rates of Infections in Safety-Net and Non–Safety-Net Hospitals **eTable 2.** Sensitivity Analyses: Comparison of the Disparity in Health Care–Associated Infection Rates Between Safety-Net and Non–Safety-Net Hospitals in the Post- vs Pre-Value-Based Incentive Program Implementation Periods
