# Supplementary material for: Association Between Federal Value-Based Incentive Programs and Health Care–Associated Infection Rates in Safety-Net and Non–Safety-Net Hospitals
Source: JAMA Netw Open. 2020 Jul 8;3(7):e209700. doi: 10.1001/jamanetworkopen.2020.9700 (PMC7344380; doi:10.1001/jamanetworkopen.2020.9700)
Supplement: Supplement. — eTable 1. Association of Value-Based Incentive Program Implementation and Health Care–Associated Infection Case Definition Changes on Reported Quarterly Rates of Infections in Safety-Net and Non–Safety-Net Hospitals eTable 2. Sensitivity Analyses: Comparison of the Disparity in Health Care–Associated Infection Rates Between Safety-Net and Non–Safety-Net Hospitals in the Post- vs Pre-Value-Based Incentive Program Implementation Periods [file jamanetwopen-3-e209700-s001.pdf]

Supplementary Online Content

Hsu HE, Wang R, Broadwell C, et al. Association between federal value-based incentive programs and health care–associated infection rates in safety-net and non–safety-net hospitals. *JAMA Netw Open.* 2020;3(7):e209700. doi:10.1001/jamanetworkopen.2020.9700

eTable 1. Association of Value-Based Incentive Program Implementation and Health Care–Associated Infection Case Definition Changes on Reported Quarterly Rates of Infections in Safety-Net and Non–Safety-Net Hospitals

eTable 2. Sensitivity Analyses: Comparison of the Disparity in Health Care–Associated Infection Rates Between Safety-Net and Non–Safety-Net Hospitals in the Post- vs Pre-Value-Based Incentive Program Implementation Periods

This supplementary material has been provided by the authors to give readers additional information about their work.

eTable 1. Association of Value-Based Incentive Program Implementation and Health Care–Associated Infection Case Definition Changes on Reported Quarterly Rates of Infections in Safety-Net and Non–Safety-Net Hospitals

	**Incidence Rate Ratio or Odds Ratio[a] (95% CI), p value**		
**Estimated change in reported HAI rate**	**Safety Net Hospitals**	**Non–Safety Net Hospitals**	**Difference Between Safety Net & Non–Safety Net Hospitals**
CLABSI per 1,000 central line-days			
Immediate change at VBIP implementation/surveillance definition revision	1.25 (1.04 – 1.49), 0.02	1.18 (1.04 – 1.34), 0.01	1.06 (0.85 – 1.31), 0.62
Slope pre-VBIP/surveillance definition revision	0.97 (0.94 – 1.00), 0.06	0.97 (0.95 – 0.99), 0.003	1.00 (0.96 – 1.04), 0.89
Slope post-VBIP/surveillance definition revision	0.98 (0.97 – 0.996), 0.009	0.99 (0.99 – 1.00), 0.32	0.99 (0.97 – 1.00), 0.16
Change in slope (post vs. pre)	1.01 (0.98 – 1.05), 0.44	1.03 (1.00 – 1.05), 0.03	0.99 (0.94 – 1.03), 0.53
CAUTI per 1,000 catheter-days			
Immediate change at VBIP implementation	0.98 (0.79 – 1.23), 0.89	1.03 (0.88 – 1.20), 0.73	0.96 (0.73 – 1.26), 0.76
Slope pre-VBIP	0.99 (0.96 – 1.01), 0.31	1.00 (0.98 – 1.02), 0.86	0.99 (0.96 – 1.02), 0.47
Slope post-VBIP	0.98 (0.97 – 1.00), 0.07	0.98 (0.97 – 0.99), 0.0004	1.01 (0.98 – 1.03), 0.65
Change in slope (post vs. pre)	1.00 (0.97 – 1.03), 0.80	0.98 (0.96 – 1.00), 0.10	1.02 (0.98 – 1.06), 0.42

©2020 Hsu HE et al. *JAMA Network Open*

eTable 1 continuation. Association of Value-Based Incentive Program Implementation and Health Care–Associated Infection Case Definition Changes on Reported Quarterly Rates of Infections in Safety-Net and Non–Safety-Net Hospitals

	**Incidence Rate Ratio or Odds Ratio[a] (95% CI), p value**		
**Estimated change in reported HAI rate**	**Safety Net Hospitals**	**Non–Safety Net Hospitals**	**Difference Between Safety Net & Non–Safety Net Hospitals**
SSI per 100 colon surgery procedures			
Immediate change at definition revision	0.33 (0.12 – 0.93), 0.04	0.76 (0.37 – 1.56), 0.46	0.43 (0.12 – 1.53), 0.19
Immediate change at VBIP implementation	2.32 (0.81 – 6.65), 0.12	1.15 (0.54 – 2.45), 0.72	2.02 (0.55 – 7.40), 0.29
Slope pre-definition revision	0.99 (0.96 – 1.03), 0.68	1.01 (1.00– 1.03), 0.12	0.98 (0.94 – 1.02), 0.31
Slope pre-VBIP/post-definition revision	1.09 (0.98 – 1.22), 0.13	1.01 (0.93 – 1.09), 0.82	1.08 (0.94 – 1.24), 0.26
Slope post-VBIP	0.99 (0.97 – 1.02), 0.59	0.99 (0.98 – 1.00), 0.15	1.00 (0.98 – 1.03), 0.78
Change in slope (post-VBIP vs. pre-VBIP/post-definition revision)	0.91 (0.81 – 1.02), 0.11	0.98 (0.90 – 1.07), 0.66	0.93 (0.81 – 1.07), 0.30
SSI per 100 abdominal hysterectomy procedures			
Immediate change at definition revision	0.59 (0.08 – 4.50), 0.61	2.32 (0.62 – 8.59), 0.21	0.25 (0.02 – 2.86), 0.27
Immediate change at VBIP implementation	0.87 (0.11 – 6.76), 0.89	0.36 (0.09 – 1.36), 0.13	2.42 (0.21 – 28.01), 0.48
Slope pre-definition change	0.98 (0.94 – 1.03), 0.41	1.01 (0.99 – 1.04), 0.30	0.97 (0.92 – 1.02), 0.22
Slope pre-VBIP/post-definition revision	1.02 (0.82 – 1.28), 0.85	0.90 (0.78 – 1.04), 0.15	1.14 (0.87 – 1.49), 0.35
Slope post-VBIP	1.03 (1.00 – 1.06), 0.10	0.99 (0.97 – 1.02), 0.61	1.03 (0.99– 1.07), 0.11
Change in slope (post-VBIP vs. pre-VBIP/post-definition revision)	1.00 (0.80 – 1.26), 0.98	1.10 (0.95 – 1.28), 0.18	0.91 (0.69-1.19), 0.48

©2020 Hsu HE et al. *JAMA Network Open*

[a]Incidence rate ratios are reported for the CLABSI and CAUTI rates. Odds ratios are reported for the SSIs.
Abbreviations: CAUTI: catheter-associated urinary tract infection; CI: confidence interval; CLABSI: central line–associated bloodstream infection; HAI: health care–associated infection; SSI: surgical site infection; VBIP: value-based incentive program.

©2020 Hsu HE et al. *JAMA Network Open*

eTable 2. Sensitivity Analyses: Comparison of the Disparity in Health Care–Associated Infection Rates Between Safety-Net and Non–Safety-Net Hospitals in the Post- vs Pre-Value-Based Incentive Program Implementation Periods

Outcome	Pre-VBIP[a] Disparity Mean IRR or OR[b] (95% CI), p-value	Post-VBIP[a] Disparity Mean IRR or OR (95% CI), p-value	Post vs. Pre-VBIP Comparison ROR (95% CI), p-value
Restriction to only "consistent reporter" hospitals[c]			
CLABSI per 1,000 central line-days (N=560)	1.23 (1.07 – 1.42), 0.005	1.15 (1.00 – 1.32), 0.04	0.93 (0.77 – 1.13), 0.49
CAUTI per 1,000 catheter-days (n=552)	1.46 (1.23 – 1.72), <0.001	1.23 (1.04 – 1.46), 0.02	0.85 (0.69 – 1.03), 0.10
SSI per 100 colon surgeries (n=567)	1.24 (1.04 – 1.48), 0.02	1.20 (1.02 – 1.41), 0.03	0.96 (0.77 – 1.20), 0.75
SSI per 100 abdominal hysterectomies (N=538)	1.14 (0.92 – 1.42), 0.23	1.40 (1.09 – 1.80), 0.009	1.17 (0.89 – 1.55), 0.26
Inclusion of only hospitals with an available DSH index to define safety-net status			
CLABSI per 1,000 central line-days (N=581)	1.23 (1.07 – 1.42), 0.004	1.15 (1.00 – 1.32), 0.04	0.93 (0.77 – 1.13), 0.47
CAUTI per 1,000 catheter-days (n=571)	1.39 (1.16 – 1.65), <0.001	1.24 (1.05 – 1.48), 0.01	0.90 (0.73 – 1.10), 0.30
SSI per 100 colon surgeries (n=581)	1.27 (1.06 – 1.50), 0.008	1.22 (1.03 – 1.43), 0.02	0.96 (0.77 – 1.20), 0.73
SSI per 100 abdominal hysterectomies (n=574)	1.15 (0.92 –1.42), 0.22	1.41 (1.10 – 1.81), 0.007	1.18 (0.89 – 1.56), 0.25

©2020 Hsu HE et al. *JAMA Network Open*

eTable 2 continuation. Sensitivity Analyses: Comparison of the Disparity in Health Care–Associated Infection Rates Between Safety-Net and Non–Safety-Net Hospitals in the Post- vs Pre-Value-Based Incentive Program Implementation Periods

Outcome	Pre-VBIP[a] Disparity Mean IRR or OR[b] (95% CI), p-value	Post-VBIP[a] Disparity Mean IRR or OR (95% CI), p-value	Post vs. Pre-VBIP Comparison ROR (95% CI), p-value
Safety-net defined as top quartile of Medicaid inpatient-days			
CLABSI per 1,000 central line-days (n=602)	1.21 (1.05 – 1.39), 0.008	1.08 (0.95 – 1.24), 0.25	0.90 (0.74 – 1.08), 0.26
CAUTI per 1,000 catheter-days (n=592)	1.34 (1.13 – 1.59), <0.001	1.19 (1.02 – 1.40), 0.03	0.89 (0.73 – 1.08), 0.24
SSI per 100 colon surgeries (n=606)	1.24 (1.05 – 1.46), 0.01	1.20 (1.03 – 1.39), 0.02	0.97 (0.79 – 1.18), 0.76
SSI per 100 abdominal hysterectomies (n=598)	1.27 (1.03 – 1.57), 0.02	1.29 (1.01 – 1.64), 0.04	0.98 (0.74 – 1.28), 0.86
Additional hospital characteristics as predictors[d]			
CLABSI per 1,000 central line-days (n=602)	1.20 (1.04 – 1.38), 0.01	1.12 (0.98 – 1.29), 0.10	0.94 (0.77 – 1.13), 0.49
CAUTI per 1,000 catheter-days (n=592)	1.19 (1.01 – 1.40), 0.04	1.06 (0.90 – 1.25), 0.46	0.89 (0.73 – 1.09), 0.28
SSI per 100 colon surgeries (n=606)	1.19 (1.01 – 1.42), 0.04	1.16 (0.98 – 1.36), 0.09	0.97 (0.78 – 1.20), 0.76
SSI per 100 abdominal hysterectomies (n=598)	1.13 (0.91 – 1.40), 0.28	1.43 (1.11 – 1.83), 0.005	1.21 (0.92 – 1.59), 0.18

[a]The pre-VBIP implementation period included data from January 1, 2013 through December 31, 2013. The post-VBIP period included data from July 1, 2017 through June 30, 2018.
[b]IRRs are reported for CLABSI and CAUTI. ORs are reported for the SSIs.
[c]Consistent reporter hospitals are defined as hospitals contributing data in both the first year (January 1, 2013 – December 31, 2013) and last year (July 1, 2017 – June 30, 2018) of the study.
[d]Additional covariates included region, hospital size (≥400 or <400 beds), and teaching status.

©2020 Hsu HE et al. *JAMA Network Open*

Abbreviations: CAUTI: catheter-associated urinary tract infection; CI: confidence interval; CLABSI: central line–associated bloodstream infection; IRR: incident rate ratio; OR: odds ratio; ROR: ratio of ratios; SSI: surgical site infection; VBIP: value-based incentive program.

©2020 Hsu HE et al. *JAMA Network Open*
